# Mango ginger (curcuma amada) inhibits collagen-induced arthritis by modulating inflammatory cytokine levels in rats

**DOI:** 10.3906/sag-2004-105

**Published:** 2020-12-17

**Authors:** Ahmet KARATAŞ, Cemal ORHAN, Mehmet TUZCU, Nurhan ŞAHİN, İbrahim Hanifi ÖZERCAN, Süleyman Serdar KOCA, Vijaya JUTURU, Kazim ŞAHİN

**Affiliations:** 1 Department of Rheumatology, School of Medicine, Fırat University, Elazığ TURKEY; 2 Department of Animal Nutrition, Faculty of Veterinary Science, Fırat University, Elazığ TURKEY; 3 Department of Biology, Faculty of Science, Fırat University, Elazığ TURKEY; 4 Department of Pathology, School of Medicine, Fırat University, Elazığ TURKEY; 5 Research and Development, OmniActive Health Technologies Inc., Morristown USA

**Keywords:** Mango ginger, inflammation, collagen-induced arthritis, rheumatoid arthritis

## Abstract

**Background/aim:**

Mango ginger (MG: curcuma amada) has antioxidant and antiinflammatory activities. The aim was to evaluate the antiarthritic potential efficacy of MG on collagen-induced arthritis.

**Materials and methods:**

Twenty-one female Wistar-albino rats were divided into three groups. Arthritis was induced by intradermal injections of type II collagen and Freund’s adjuvant. MG extract was orally administered starting from the first collagen injection. TNF-α, IL-6, IL-17, obestatin, sclerostin, and DKK-1 serum levels were determined, and perisynovial inflammation and cartilage-bone destruction in the paws were histologically evaluated. Moreover, joint tissue TNF-α, IL-17, NF-κB, and COX-2 levels were analyzed.

**Results:**

TNF-α, IL-17, IL-6, and DKK-1 serum levels were increased, and obestatin and sclerostin serum levels were decreased in the arthritis group compared to the control group. However, MG supplements decreased TNF-α, IL-17, IL-6, and DKK-1 serum levels and increased obestatin and sclerostin serum levels. Similarly, while collagen injection increased tissue TNF-α, IL-17, NF-κB, and COX-2 levels, MG decreased TNF-α, IL-17, and NF-κB levels. Moreover, MG ameliorated perisynovial inflammation and cartilage-bone destruction in the paws.

**Conclusion:**

MG ameliorates arthritis via actions on inflammatory ways and wingless (Wnt) signaling pathway. These results suggest that MG may have a considerable potential efficacy for the treatment of rheumatoid arthritis.

## 1. Introduction

Rheumatoid arthritis (RA), a chronic and progressive autoimmune disease, is characterized by inflammation triggered by genetic and environmental factors [1]. The most common clinical finding is joint involvement in RA. The inflammatory process begins with the interaction of innate immunity (macrophage, neutrophil, dendritic cell, etc.) with acquired immunity (T and B lymphocytes) and with fibroblast-like synoviocytes. This process becomes chronic with the interaction of some signal transduction pathways, the release of autoantibodies (rheumatoid factor and anti-cyclic citrulline peptides), and proinflammatory cytokines (tumor necrosis factor [TNF]-α, interleukin [IL]-1β, IL-6, and IL-17) [2,3].

Wingless (Wnt) signaling pathway plays a very important role in the regulation of the cell’s proliferation and differentiation as well as bone homeostasis. Wnt pathway expresses target genes and leads to the synthesis of different proteins. Thus, cellular events are controlled such as inhibition of cell proliferation, differentiation, apoptosis, and angiogenesis [4,5]. Wnt pathway plays a crucial role in RA pathogenesis. Sclerostin and Dickkopf (DKK)-1 are extracellular proteins that inhibit this pathway [u292b,7].

Nuclear factor kappa B (NF-κB) activation plays an important role in the initiation and maintenance of inflammation in RA. NF-κB causes the release of some mediators and enzymes by inducing transcription of genes involved in inflammation, pain, and oxidative stress [8].

Obestatin is an anorectic peptide encoded by the same gene of the ghrelin hormone. Obestatin is synthesized in many tissues such as the stomach, small intestine, hypothalamus, and pituitary. It has the opposite effect of ghrelin and suppresses appetite and reduces weight gain [9]. It has been shown that obestatin levels may be associated with some inflammatory markers in systemic inflammatory diseases such as RA [10].

Treatment of RA is difficult because of the uncertainty of underlying pathogenetic mechanisms and the variety of clinical findings. Various herbal formulations have been used since ancient times for the alternative treatment research. Mango ginger (MG) is used as herbal medicine to treat inflammation. MG has been reported to have many effects such as anti-oxidant, anti-cancer, analgesic, and antiinflammatory [11]. Therefore, this study was planned to demonstrate whether MG has antiinflammatory effects on an experimental arthritis model, collagen-induced arthritis (CIA).

## 2. Material and methods

### 2.1. Animals

Twenty-one female Wistar albino rats (8 weeks old; 200–220 g) were purchased from Animal Experimental Unit of Fırat University. Rats were maintained on a 12 h light, 12 h dark cycle at a temperature of 22 ± 3 °C and a relative humidity of 55% ± 5%. Animals received the standard pellet diet as described previously [12] and water ad libitum. The experimental procedures were approved by the Ethical Committee of Animal Care of Firat University (2016/15-147).

### 2.2. Study groups and applications

Rats were divided randomly into 3 groups (n = 7) as control, arthritis+placebo, and arthritis+MG supplement groups. Type 2 collagen from the chicken sternum (Sigma Aldrich, St. Louis, USA) was dissolved in 0.1 M acetic acid (to reach 1 mg/mL concentration of type 2 collagen). The collagen solution was emulsified in an equal amount of incomplete Freund’s Adjuvant (Difco Laboratories, Detroit, USA) [13]. In arthritis + placebo and arthritis + MG groups, this solution was given intradermally (total: 200 μg to each rat) to the rats from tail dorsal (100 μg to each rat) and hindpaw (50 μg to each hindpaw), for arthritis induction. Seven days after the first injection, booster injection (100 μg to each rat) was applied in tail dorsal. Rats in the third (arthritis+MG) group were orally administered MG at a dose of 50 mg/kg/day (OmniActive Health Technologies, Inc., Morristown, NJ, USA) starting from the first collagen injection to 33rd day. The dose was determined based on Ramachandran et al. [14]. MG was administered by gavage, and saline solution was used as a vehicle.

All rats were evaluated daily for arthritis development and clinical arthritis scoring. Clinical arthritis score was assessed by scoring 0–4 points [15].

All rats were sacrificed by decapitation on the 33rd day and the study was terminated. Blood samples of the rats were taken and centrifuged at 5000 rpm for 5 min. The harvested sera were stored at –20 °C until the day of analysis. In all groups, the hind paws were excised rapidly from sacrificed rats for later histopathological and Western blot analysis. Tissue samples were fixed with 10% formalin solution for further pathological analysis. The tissues for Western blot analysis were quickly frozen at –80 °C.

### 2.3. Histopathological analysis

Tissue samples, those fixed with formalin solution, were decalcified with 10% nitric acid (30 days), then paraffin blocks were prepared. Sections from the blocks were stained with Hematoxylin-Eosine (H&E stain). Sections were examined by a specialist pathologist under the light microscope ×20, ×40, ×100, ×200, and ×400 magnifications in the pathology laboratory to assess inflammatory cell infiltration, pannus formation, and destruction of the bones around the joints. Histopathological scoring was performed (0– 4 score) [16].

### 2.4. Laboratory analyses

Routine serum laboratory assays (glucose, alanine aminotransferase (ALT), aspartate aminotransferase (AST), urea, creatinine, and lipid profiles) were determined by the biochemical analyzer (Samsung LABGEO PT10, Samsung Electronics Co, Suwon, Korea). Serum IL-17, IL-6, TNF-α, DKK-1, sclerostin and obestatin levels were analyzed by using commercially available enzyme-linked immunosorbent assay (ELISA) kits according to the recommended procedure by the manufacturer’s instructions (Cayman Chemical, Ann Arbor, MI, USA).

### 2.5. Western blotting

Levels of TNF-α, IL-17, NF-κB, and COX-2 in joint tissue samples were analyzed using Western blot technique. In accordance with the purpose, the hind paws were quickly harvested and frozen at –80 °C. The joint tissue homogenates were prepared in ice-cold lysis buffer containing 50 mM Tris-HCl (pH, 8.0), 5 mM EDTA, 0.26% sodium deoxycholate, 1% Triton X-100, 150 mM NaCl, 50 mM sodium fluoride, 10 mM b-glycerophosphate, 0.1 mM sodium orthovanadate, 50 μg/ml phenylmethylsulfonyl fluoride, and 10 μg/mL leupeptin. The prepared homogenates were incubated on ice for 40 min [16].

Sodium dodecyl sulfate-polyacrylamide gel electrophoresis sample buffer containing 2% b-mercaptoethanol was added to the supernatant. Equal amounts of protein (50 μg) were electrophoresed and then transferred to nitrocellulose membranes (Schleicher and Schuell Inc., Keene, NH, USA). Nitrocellulose blots were each washed in phosphate-buffered saline (PBS) for 5 min and blocked with 1% bovine serum albumin in PBS for 1 h prior to administration of the primary antibody. Antibodies against TNF-α, IL-17, NF-kB, COX-2, and IL-17 (Abcam, Cambridge, UK) were diluted (1: 1000) in the same buffer containing 0.05% Tween-20. The nitrocellulose membrane was incubated with protein antibody overnight at 4 °C. The blots were washed and the turbot peroxidase conjugated goat antimouse IgG (Abcam, Cambridge, UK). Specific binding was detected using H2O2 and diaminobenzidine, as substrates. Protein loading was checked using a monoclonal mouse antibody against β-actin antibody (A5316; Sigma Aldrich, St. Louis, MO, USA). Blots were performed at least three times to confirm the reproducibility of the results. Bands were analyzed densitometrically using an image analysis system (Image J; National Institute of Health, Bethesda, USA).

### 2.6. Statistical analysis

The sample size of the present study was seven per group and it was calculated based on a power of 85% and a P value of 0.05 (GPower 3.1). All analyses were performed using the general linear model procedure. Differences among groups were analyzed by Fisher’s post hoc test and Mann–Whitney U test for categorical and continuous variables, respectively.

## 3. Results

Swelling and erythema were observed around 13 to 15 days after the first injection of collagen. The 15th day arthritis score was significantly higher in arthritis and MG groups than the control group (P < 0.001 and P < 0.05, respectively; Table 1). The 15th day arthritis scores were decreased by 36% in the MG group when compared to the arthritis group (P ˃ 0.05; Table 1). Arthritis scores of the 33rd day were decreased by 44% in the MG group compared to the arthritis group (P < 0.001). MG supplement ameliorated the clinical findings of arthritis (Table 1).

**Table 1:** The effect of mango ginger extract on arthritis scores, inflammation score, and biochemical parameters in rats with collagen-induced arthritis.

Parameters	Control	Arthritis	Mango ginger
15th day Arthritis Score	-	2.00 ± 0.38	1.29 ± 0.29
33rd day Arthritis Score	-	3.57 ± 0.20	2.00 ± 0.38##
Inflammation Score	-	4.43 ± 0.62	2.64 ± 0.46##
Blood glucose, mg/dL	97.57 ± 3.53	100.71 ± 3.43	98.43 ± 3.40
ALT, U/L	49.71 ± 5.64	50.86 ± 4.18	48.00 ± 2.10
AST, U/L	324.29 ± 19.11	326.86 ± 13.54	323.43 ± 15.09
Creatinine, mg/dL	1.72 ± 0.19	1.78 ± 0.10	1.74 ± 0.07
Cholesterol, mg/dL	94.43 ± 5.40	96.14 ± 6.38	93.86 ± 4.50
Triglycerides, mg/dL	115.29 ± 5.88	118.14 ± 5.48	116.29 ± 5.81
HDL-c, mg/dL	28.14 ± 2.01	26.71 ± 1.86	26.71 ± 2.04
LDL-c, mg/dL	41.29 ± 3.48	42.86 ± 2.32	42.00 ± 3.63

ALT: Alanine aminotransferase; AST: Aspartate aminotransferase; HDL-c High-density lipoprotein-cholesterol; LDL-c: Low-density lipoprotein-cholesterol.The data presented as a mean ± standard deviation. Mean values within the same row with are statistically different for

### 3.1. Cytokine levels

Serum IL-6, TNF-α, and IL-17 levels were higher in the arthritis group than the control group (P ˂ 0.001 for all three). On the other hand, serum TNF-α, IL-6, and IL-17 levels were lower (36%, 29%, and 41%, respectively) in the MG group when compared with the arthritis group (P < 0.001 for all three) (Table 2).

**Table 2:** The effect of mango ginger extract on serum IL-6, TNF-α, obestatin, sclerostin, and DKK-1 levels in rats with collagen-induced arthritis.

Parameters	Control	Arthritis	Mango ginger
Serum IL-6, pg/mL	11.04 ± 0.75	39.14 ± 2.83***	27.71 ± 1.52*** ##
Serum IL-17, pg/mL	22.43 ± 1.66	60.29 ± 3.22***	35.86 ± 2.73** ###
Serum TNF-α, pg/mL	24.71 ± 1.73	52.43 ± 2.21***	33.57 ± 1.60** ###
Obestatin, pg/mL	279.43 ± 14.43	118.71 ± 4.82***	143.71 ± 5.30***
Sclerostin, ng/mL	0.49 ± 0.03	0.22 ± 0.01***	0.30 ± 0.02*** #
DKK-1, pg/mL	643.57 ± 32.49	1700.71 ± 25.27***	1318.29 ± 25.80*** ###

IL: Interleukin; TNF-α: Tumor necrosis factor alpha; DKK: Dickkopf - related protein. Data were presented as mean ± standard deviation. Mean values within same row with are statistically different for **P < 0.01, ***P < 0.001 as compared with control group; #P < 0.05, ##P < 0.01,

### 3.2. DKK-1 and sclerostin levels

Serum DKK-1 levels were higher in the arthritis group compared to the control group (P < 0.001). Serum DKK-1 levels were 23% lower in the MG group when compared to the arthritis group (P < 0.001). Serum sclerostin levels were 55% lower in the arthritis group compared to the control group (P < 0.001). Serum sclerostin levels were 36% higher in MG group when compared with the arthritis group (P < 0.05) (Table 2).

### 3.3. Obestatin levels

Serum obestatin levels were 58% lower in the arthritis group when compared to the control group (P < 0.001). Serum obestatin levels were 21% higher in the MG group when compared with the arthritis group, but there was no statistically significant difference (Table 2; P ˃ 0.05).

### 3.4. Western blot analyzes

**Figure 1:**
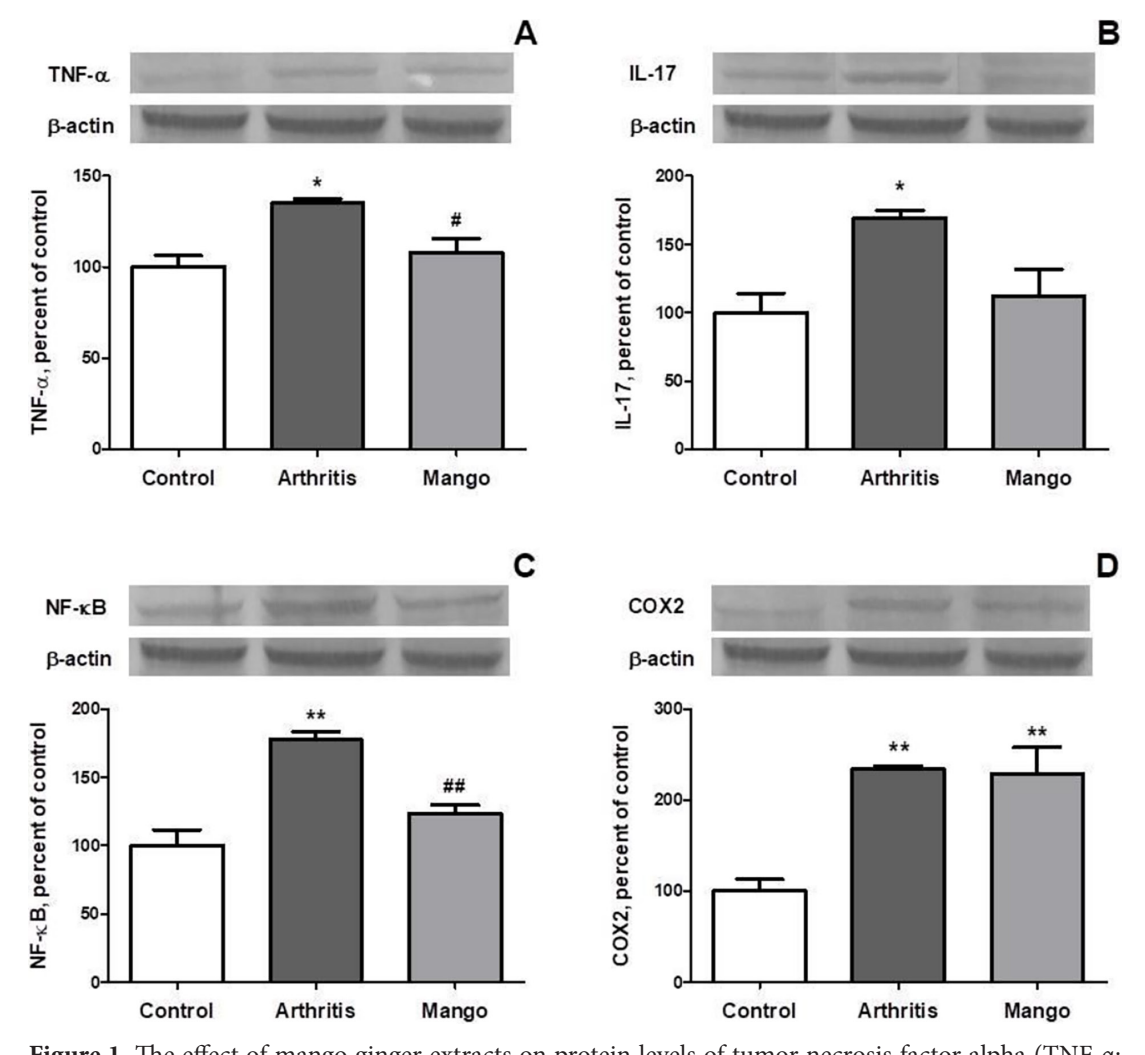
The effect of mango ginger extracts on protein levels of tumor necrosis factor alpha (TNF-α; Panel A), interleukin 17 (IL-17; Panel B), nuclear factor-kappa B (NF - kB; Panel C), and cyclooxygenase 2 (COX2; Panel D) in the study groups. The intensity of the bands was quantified by densitometric analysis and β-actin was included to ensure equal protein loading. Data are expressed as percent of the control value. Blots were repeated at least 3 times. *P < 0.05, **P < 0.01, as compared with the control group; #P < 0.05, ##P < 0.01 as compared with the arthritis group.

The tissue NF-κB levels were 77% higher in the arthritis group when compared with the control group (Figure 1C). NF-κB levels were decreased by 30% in the MG group when compared with the arthritis group (P ˂ 0.05). COX-2 levels were 133% and 128% higher in arthritis and MG group compared with the control group, respectively. There was no difference between arthritis and MG groups in terms of COX-2 levels (P ˃ 0.05) (Figure 1D). The joint tissue TNF-α levels were increased by 35% in the arthritis group when compared to the control group (Figure 1A). The joint tissues TNF-α levels were decreased by 20% in the MG group when compared with the arthritis group (P ˂ 0.05). The joint tissue IL-17 levels were 69% higher in the arthritis group when compared with the control group (P ˂ 0.05). The joint tissue IL-17 levels were 33% lower in the MG group when compared with the arthritis group (P < 0.05) (Figure 1B).

### 3.5. Histopathological evaluation

**Figure 2:**
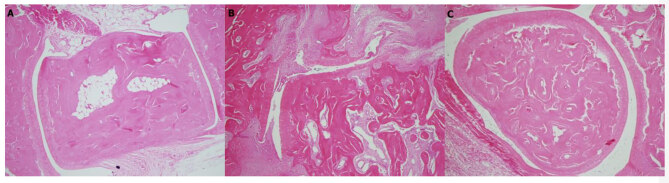
The effect of mango ginger extracts on histopathological changes (H & E × 40) in the study groups. Normal joint structure in the control group (A). Marked perisynovial inflammatory cell infiltration and cartilage–bone destruction in the arthritis group (B). Decreased perisynovial inflammation and synovial hyperplasia in the mango ginger group (C).

In the histopathological evaluation of the joint tissue samples taken from rats, marked perisynovial inflammatory cell infiltration and cartilage-bone destruction in the arthritis group were observed (Figure 2A and 2B). There was a decrease in the inflammatory cell infiltration and bone-cartilage destruction in the MG group (Figure 2C). The inflammation score was decreased by 40% in the MG group when compared with the arthritis group (P ˂ 0.001).

## 4. Discussion

Rheumatoid arthritis is a disease that causes malfunctioning of the joints as a result of inflammation and proliferation of the synovial membrane. If not properly treated, it can lead to serious deformities and disabilities. However, cure or remission cannot be reached with available therapeutic agents in all RA patients. Moreover, medical treatment agents used in RA may have serious side effects such as osteoporosis, hypertension, diabetes mellitus, infection, and malignancy [17]. For this reason, the new treatment option which is effective, reliable, and has a low side effect is a necessity in RA.

Mango ginger is within the Zingiberaceae family and *Curcuma* genus; it usually grows in tropical regions. MG is also known as *Curcuma amada* . MG is used as a traditional treatment modality in the world because of its many useful biological activities such as antimicrobial, antiinflammatory, antioxidant, antitumor, hypotriglyceridemia, antiaggregation, and analgesia [18]. In an experimental arthritis study, MG has been shown to reduce joint swelling, arthritic index, white blood cell count, and erythrocyte sedimentation rate [19]. In another experimental study, Mujumdar et al. [20] have demonstrated that MG has dose-dependent antiinflammatory activity in acute and chronic administration. In our study, there was a decrease in arthritis scores in the MG group.

Activated T cells play an important role in RA pathogenesis. Activation of T cells induces monocytes, macrophages, and synovial fibroblasts and increases the production of proinflammatory cytokines such as TNF-α, IL-6, and IL-1. Activated T cells are responsible for going on the inflammatory process. Th17 cells play a central role in RA pathogenesis by producing IL-17 and TNF-α. IL-17 stimulates synovial fibroblasts to produce IL-6 and monocyte-macrophages to produce TNF-α. RANKL (receptor activator of NF - kB ligand) is expressed on the Th17 cells surface, so thus the conversion of nonresorptive osteoclasts to resorptive osteoclasts is induced. As a result, osteoclastogenesis is induced and bone-cartilage destruction occurs [21]. TNF-α is one of the main cytokines that play a role in the pathogenesis of RA. TNF-α blockade has been shown to reduce bone-cartilage destruction [22,23]. Consequently, therapeutic agents blocking TNF-α have been developed and these agents are widely used in routine practice to treat RA.

Interleukin-6 activates neutrophils, T cells, and osteoclasts and increases the levels of acute phase proteins, and thus IL-6 leads to joint destruction in RA [24]. High IL-6 levels in patients with RA have been shown to correlate with radiological joint destruction [2]. In the current study, the serum levels of cytokines such as TNF-α, IL-6, and IL-17 tissue levels of TNF-α, and IL-17 were decreased in the group treated with MG. These cytokines are accepted to orchestrate the pathogenesis of RA.

In RA, synovial fibroblasts produce inflammatory and proliferative mediators. The Wnt pathway has an important function in the activation of synovial fibroblasts [4]. DKK-1 is secreted from synovial fibroblasts in response to inflammatory conditions. DKK-1 has an important role in bone biology by suppressing osteoblastic activity [25]. It also inhibits the Wnt signaling pathway and thus reduces the formation of osteoprotegerin. Eventually, it causes an increase in osteoclastic activity [26]. The DKK-1 expression has been shown to increase in the experimental arthritis model [25]. Caetano –Lopes et al. [27] have shown that DKK-1 expression in RA increases the bone fragility. Besides, it is known that TNF-α and IL-6 increase DKK-1 levels and thus they inhibit new bone formation [28]. The preventive effects of antiTNF therapies on bone damages are a consequence of their suppressive action on serum levels of DKK-1 [reviewed in 29]. In our study, serum DKK-1 levels increased in the arthritis group and decreased in the MG group.

Sclerostin is a protein secreted from osteocytes encoded by the SOST gene. It is an antagonist of the Wnt pathway like DKK-1 and increases bone destruction by suppressing the osteoblastic process [26]. Studies done with sclerostin in RA have different results. In one study, improvement of bone damage with sclerostin inhibitors has been observed [30]. Different studies have shown that there is no significant change in serum sclerostin levels in RA compared to healthy controls [31,32]. Wehmeyer et al. [33] have shown that the inhibitors of sclerostin induce joint destruction by triggering TNF–α-dependent inflammatory processes and thus lead to poor outcomes, in patients with RA. In our study, sclerostin levels increased in the MG group compared to the arthritis group. These results suggest that MG affects Wnt pathway.

Nuclear factor kappa B plays an important role in the regulation of the immune system by transcriptional activation of certain proinflammatory cytokines. In particular, activation of the classical NF-κB signaling pathway induces the osteoclastic process [29]. Cytokine production in patients with RA is dependent on NF-κB [34]. In our study, NF-κB activation reduced with MG treatment.

In RA, COX-2 is released from various cells, such as monocytes, macrophages, synovial cells and fibroblasts which are associated with inflammatory response, and it causes joint damage [35]. The inhibition of COX-2 reduces synovial inflammation and thus prevents joint damage. Furthermore, the COX-2 expression is regulated by the NF-κB pathway. COX-2 levels were not sufficiently reduced with MG treatment, in our study. This may be related to the short duration of treatment or that selected MG dose may be low [36,37]. In CIA model, MG inhibits several inflammation-related ways such as NF-κB binding activity, NF-κB dependent expression of cytokines (TNF-α, IL-6, and IL-17), T and B lymphocytes, and macrophages in synovial cells. It may be concluded that MG contains a number of bioactive divergent substances which inhibit inflammatory and osteoclastic activity.

Obestatin is associated with the inflammatory process in some diseases as well as metabolic process. Obestatin has antiinflammatory and antioxidant effects. It has been shown that it decreases some proinflammatory cytokine levels and inhibits inflammatory pathways [38,39]. Koca et al. [10] have shown no significant difference in obestatin levels between RA and healthy controls. In the same study, it was shown that obestatin levels were higher in patients with RA than Behçet’s disease [10]. In another study, it has been shown that obestatin treatment reduces the expression of vascular cell adhesion molecule-1 (VCAM - 1) [40]. On the other hand, Alexandridis et al. [41] have shown that serum obestatin levels are decreased in active inflammatory bowel disease. In our study, obestatin level was decreased in arthritis while MG treatment increased serum obestatin level. Although controversial results are reported in different studies, all these data we have obtained suggest that the mechanisms underlying anti-arthritic effects of MG may be related to obestatin.

As a result, in experimental arthritis model, MG treatment decreases the levels of cytokines such as TNF-α, IL-6, and IL-17. It also depletes NF-kB expression and the level of serum DKK-1, which is the Wnt pathway inhibitor. On the other hand, MG increases the serum levels of sclerostin and obestatin. These results reveal that MG applications alter several pathways that are associated with RA pathogenesis. These results suggest that MG therapy may be effective in RA treatment by suppressing inflammatory and osteoclastic pathways.
